# Explore the Mechanism of Xiaoyaosan for Treatment of Bipolar Disorder Based on Network Pharmacology, Experimental Validation and Molecular Docking

**DOI:** 10.1002/cns.71179

**Published:** 2026-09-28

**Authors:** Man Chen, Nana Ding, Xingmin Qiu, Lan Li, Yali Guo, Rongyanqi Wang, Xiaowei Mo, Tan Zou, Shangzhen Yu, Xiaojuan Li, Jiaxu Chen

**Affiliations:** ^1^ Affiliated Jiangmen Traditional Chinese Medicine Hospital of Jinan University Jiangmen People's Republic of China; ^2^ Guangzhou Key Laboratory of Formula‐Pattern of Traditional Chinese Medicine, School of Traditional Chinese Medicine Jinan University Guangzhou People's Republic of China; ^3^ Integrated Chinese and Western Medicine Postdoctoral Research Station Jinan University Guangzhou People's Republic of China; ^4^ Henan Province Hospital of Traditional Chinese Medicine Zhengzhou People's Republic of China; ^5^ School of Chinese Medicine Beijing University of Chinese Medicine Beijing People's Republic of China

**Keywords:** bipolar disorder, mechanism, molecular docking, network pharmacology, Xiaoyaosan

## Abstract

**Aim of the Study:**

Xiaoyaosan (XYS) has significant anti‐inflammatory effects and is widely used for treating depression. However, the multitarget mechanism of XYS in the treatment of bipolar disorder (BD) remains unexplored. In this study, network pharmacology, molecular docking, and experimental validation were integrated to elucidate the mechanisms of XYS and its therapeutic efficacy in BD.

**Methods:**

First, network pharmacology was employed to elucidate the anti‐BD mechanism of XYS. Second, the therapeutic effects of XYS against BD were investigated in a BD mouse model induced by Ank3 gene knockdown combined with chronic social defeat stress (CSDS). Finally, the targets from these predictions were validated in a series of experiments and molecular docking.

**Results:**

First, on the basis of network pharmacology, 120 predicted intersection targets between the targets of XYS and BD‐related targets were identified. The key core targets of the anti‐BD activity of XYS were STAT3, AKT1, IL‐6, and MAPK3. Thus, inflammation pathways might play a critical role in the anti‐BD effects of XYS. Second, XYS clearly inhibited BD activity, which was manifested by a significant alleviation of manic hyperactivity behavior and a significant improvement in depressive hyperactivity behavior in BD model mice. Additionally, the up‐regulation of STAT3, AKT1, IL‐6, and MAPK3 was improved by XYS intervention in a BD mouse model. Moreover, molecular docking results also revealed that XYS showed strong stable binding to key core targets from network pharmacological predictions.

**Conclusions:**

XYS can markedly improve BD through modulation of the expression of the key multitargets STAT3, AKT1, IL‐6, and MAPK3 in inflammation pathways. These novel findings elucidate the anti‐BD mechanism of XYS from the perspective of multiple targets and pathways.

## Graphical abstract

This study evaluates Xiaoyaosan (XYS) for bipolar disorder (BD) using network pharmacology and experimental validation. Molecular docking reveals interactions between active XYS compounds and potential core targets. Animal experiments confirm XYS alleviates BD‐like behaviors in mice by suppressing core targets, elucidating its multi‐target mechanism and therapeutic potential.

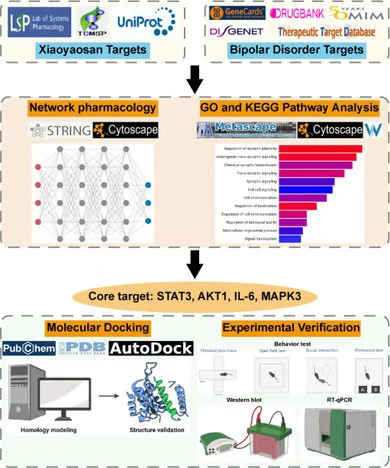

**Abbreviations**
AAV: adeno‐associated virus
AKT1: RAC‐alpha serine/threonine‐protein kinase
BD: bipolar disorder
BP: biological process
CC: cellular components
CSDS: chronic social defeat stress
DC: degree centrality
EPMT: elevated plus‐maze test
FST: forced swim test
GO: gene Ontology
IL‐6: interleukin‐6
KEGG: Kyoto encyclopedia of genes and genomes
LiCO_3_
: lithium carbonate
MAP K3: mitogen‐activated protein kinase 3
MF: molecular functions
NF‐κB: nuclear factor Kappa‐light‐chain‐enhancer of activated B cells
OB: oral bioavailability
OFT: open field test
PPI: protein–protein interaction
SIT: social interaction test
STAT3: signal transducer and activator of transcription 3
TCM: traditional Chinese medicine
TCMSP: traditional Chinese medicine systems pharmacology
TST: tail suspension test
XYS: xiaoyaosan

## 1 Introduction

Bipolar disorder (BD) is a complex, chronic psychiatric disorder that consists of recurrent episodes of mood disturbances—depressive episodes—together with manic or hypomanic phenomena [1]. Manic episodes typically involve symptoms such as elevated mood, irritability, increased physical activity, and agitation. Conversely, depressive episodes involve low mood, depression, and decreased physical activity. BD is characterized by high recurrence rates, high disability rates, and low cure rates. BD has a global lifetime prevalence of 2.4% [2], with a suicide rate among patients with depressive episodes of 11%, which is significantly higher than that observed among patients with major depressive disorder [3, 4]. Furthermore, the worldwide burden of BD has continuously increased. For example, BD patients cost approximately 5.8 billion euros per year in Germany [5], and the total cost for BD patients in the United States over 10 years has been estimated at 204 billion dollars [6]. Pharmacotherapy, psychosocial therapy, and physical therapy are among the various pharmacologic and therapeutic treatment options for BD. Lithium carbonate (LiCO_3_) was discovered in 1981 [7], and is currently the first‐line pharmacological treatment for BD. While it is effective at alleviating the manic and depressive symptoms of BD, persistent administration invariably induces multiple side effects, including cognitive dysfunction [8], lithium toxicity [9], tardive dyskinesia [10], and metabolic disorder syndrome [11]. Many patients abandon treatment because of these adverse effects, underscoring the critical need for better therapeutics to improve BD management.

The causes of BD are presumed to be multifactorial and include a combination of social, psychological, familial, and genetic risk factors. Among these, genetic factors are thought to be among the most important risk factors for developing BD [12, 13], with 60%–80% of cases designated as heritable. In recent years, the Ank3 gene has been recognized as the main susceptibility gene for BD, and it is involved mainly in increasing the genetic predisposition to BD [14, 15]. Ankyrin‐G (Ank3) is a linker‐associated protein located principally in the nodal regions of nerve fibers. It controls action potential propagation via Nav and Kv channels, which are required for neuronal excitability and the stability of neural circuits [16]. Recent reports have indicated that specific mutations in single‐nucleotide polymorphism (SNP) loci, including rs10994336, rs1938526, rs10994415, and rs9804190, within the Ank3 gene serve as independent risk factors for BD and are capable of affecting neuronal excitability [17]. Gene targeting has been used to conditionally knock out Ank3 in the pyramidal neurons of the brains of mice, resulting in the generation of severe lithium‐responsive manic‐like behaviors that are in agreement with the data presented herein [18]. Additionally, under combined conditions of chronic social defeat stress (CSDS), these mice also manifested unique depressive‐like behaviors that closely resembled the behavioral phenotypes found in BD patients, providing a robust model for investigating the mechanisms underlying BD pathogenesis and for the design of new drugs.

Xiaoyaosan (XYS), as a traditional Chinese herbal formula, comes from the “Taiping Huimin Heji Jufang” dating back to the Song Dynasty. It consists of eight Chinese herbal medicines: Chai Hu (*Bupleurum Chinense DC*.), Dang Gui (*Angelica sinensis (Oliv.) Diels*), Bai Shao (*Paeonia lactiflora Pall*.), Bai Zhu (*Atractylodes macrocephala Koidz*.), Fu Ling (*Smilax glabra Roxb*.), Gan Cao (*Glycyrrhiza uralensis Fisch*.), Bo He (*Mentha canadensis L*.), and Sheng Jiang (*Zingiber officinale Roscoe*). XYS is renowned for its efficacy, including soothing the liver Qi, resolving depression, strengthening the spleen, and replenishing blood; it is primarily utilized to treat syndromes associated with liver qi stagnation and spleen dysfunction. This syndrome is closely related to emotional and behavioral disorders such as depression and anxiety. In a cohort study, Shuping Chen et al. [19] examined the traditional Chinese medicine in BD patients. After analyzing the results, they reported that the combination of carbamazepine and Jia‐Wei‐Xiaoyaosan significantly alleviated both hypomanic and depressive episodes overall in patients with BD, reducing the severity of depressive episodes and enhancing the overall quality of life of patients. These results indicated that XYS could be a beneficial treatment choice for BD. The mechanisms of action and bioactive compounds of XYS for BD treatment are still unclear and require further exploration.

Consequently, network pharmacology and molecular docking were employed to screen the potential core targets and biologically active constituents of XYS in the intervention of BD. Furthermore, a BD model was constructed by conditionally knocking down the Ank3 gene in the mouse hippocampus combined with CSDS to verify the screening results. This study provides new perspectives on the mechanism of action and material basis of XYS in the treatment of BD.

## 2 Materials and Methods

### 2.1 Network Pharmacology Analysis

#### 2.1.1 Screening of Compounds and Prediction of Targets in XYS

Information on the biologically active constituents and corresponding targets of the eight medicines in XYS was extracted from the Traditional Chinese Medicine Systems Pharmacology (TCMSP) database [20]. The screening process applied the following filtering criteria: oral bioavailability (OB) ≥ 30% and drug likeness (DL) ≥ 0.18. To standardize target names, we employed the UniProt database [21] to ensure uniform formatting.

#### 2.1.2 Construction of a Network Diagram Representing the Relationship Between Drugs and Biologically Active Constituents

A network diagram of the specific active compounds and their targets was constructed using Cytoscape, one of the most frequently used software programs for network visualization [22]. The biologically active constituents of the drug were collected and imported into Cytoscape 3.7.2 for visualization. Using Network Analyzer, we calculated three key topological parameters of the interaction network: degree centrality (DC), closeness centrality (CC), and betweenness centrality (BC). This analysis facilitated the exploration of the relationships among the biologically active constituents, drugs, and potential targets of XYS.

#### 2.1.3 Collection of Disease Targets

Using “bipolar disorder” as the search keyword, we accessed several databases to identify disease targets, including OMIM [23], GeneCards [24], Drugbank [25], TTD [26], and DisGeNET [27]. The retrieved targets were standardized using the UniProt database, and duplicate values were removed across the five disease databases. This process facilitated the creation of a comprehensive dataset of targets associated with BD.

#### 2.1.4 Acquisition of Common Targets for XYS and BD

The identified targets were subsequently entered into the online tool Venny 2.1.0 to generate Venn diagrams, which enabled the identification of common targets between XYS and BD.

#### 2.1.5 Construction of a Protein–Protein Interaction (PPI) Network Diagram

The common targets were uploaded to the STRING 11.0 database [28]. To create PPI network diagrams, we chose “ *Homo sapiens* ” as the species, excluded disconnected nodes, and established the minimum required interaction score at a high confidence level of 0.900. The findings were loaded into Cytoscape 3.7.2 to generate a PPI network diagram. Protein interaction data were weighted and integrated, and DC values were calculated using the Network Analyzer tool. Higher DC values were considered indicative of key therapeutic targets.

#### 2.1.6 GO Enrichment and KEGG Pathway Analyses

The potential targets were analyzed and subjected to GO and KEGG pathway analyses using Metascape [29]. These analyses aimed to examine the biological processes (BP), molecular functions (MF), and cellular components (CC) associated with the identified targets. For both the GO and KEGG analyses, we set the minimum overlap at 3, the cut‐off *p* value at 0.01, and the minimum enrichment threshold at 1.5. The results were sorted in ascending order according to the log *p* value. A corresponding bubble diagram was then generated using the bioinformatics server for visualization. To ensure robustness, the minimum gene set for enrichment was set to no less than 10, and the top 20 enriched terms are shown. Furthermore, to visualize the relationships among the compounds, targets, and pathways, a compound‐target‐pathway network was constructed and evaluated using Cytoscape 3.7.2.

#### 2.1.7 Molecular Docking

Molecular docking was performed to study the interactions between core targets and their respective bioactive components. The structures of the compounds of interest were retrieved from the PubChem database in SDF format. These structures were subsequently converted to MOL2 format files using Chem3D software. The protein structures were obtained in PDB format from the RCSB database [30]. The ligand and solvent molecules were deleted with PyMOL software. Hydrogen atom addition, charge calculations, and atomic type assignment were performed using AutoDock Tools 1.5.6. Afterwards, the output data were converted and saved in the pdbqt format. Molecular docking was subsequently conducted with AutoDock Vina 1.1.2 [31], and the docking results were visualized and analyzed using PyMOL 2.3.0.

### 2.2 Animal Experimental Validation

#### 2.2.1 Reagent

The XYS powder was purchased from Hunan Jiuzhitang Co. Ltd. (Changsha, China). This powder consists of eight medicinal herbs based on the theoretical foundation of Traditional Chinese Medicine: Chai Hu (*Bupleurum Chinense DC*.), Dang Gui (*Angelica sinensis (Oliv.) Diels*), Bai Shao (*Paeonia lactiflora Pall*.), Bai Zhu (*Atractylodes macrocephala Koidz*.), Fu Ling (*Smilax glabra Roxb*.), Gan Cao (*Glycyrrhiza uralensis Fisch*.), Sheng Jiang (*Zingiber officinale Roscoe*), and Bo He (*Mentha canadensis L*.), at a ratio of 5:5:5:5:5:5:4:1. For this study, XYS was dissolved in deionized water, and the mice were administered an intragastric dose of 0.658 g/kg/day for 4 weeks [32, 33]. LiCO_3_ (Sigma‐Aldrich, St. Louis, Missouri) dissolved in deionized water was used as a positive control and was administered at a dosage of 85 mg/kg per day [34]. The mice in the NC and BD groups were given deionized water (0.1 mL/10 g body weight).

#### 2.2.2 Sample Preparation

The traditional Chinese medicine compound XYS was provided by Hunan Jiuzhitang Co. Ltd. and prepared according to the “Chinese pharmacopeia 2020 Edition” for Xiaoyao Pill (Pharmacopeia 2020 edition of the People's Republic of China, China Medical Science and Technology Press, 2020). The composition and preparation methods were as follows: *Bupleurum Chinense DC*. 100 g, *Angelica sinensis (Oliv.) Diels* 100 g, *Paeonia lactiflora Pall*. 100 g, *Rhizoma atractylodis* 100 g, *Smilax glabra Roxb*. 100 g, *Glycyrrhiza uralensis Fisch*. 100 g, *Zingiber officinale Roscoe* 80 g, and *Mentha canadensis L*. 20 g. The following extraction methods were applied: 50 g of *Bupleurum Chinense DC*. and *Angelica sinensis (Oliv.) Diels*, 100 g of *Mentha canadensis L*. and *Zingiber officinale Roscoe*, were used to extract essential volatile oils. Decoction residues of *Rhizoma atractylodis* and *Smilax glabra Roxb*. were boiled twice in water for 2 h each time. The two decoctions were combined, filtered, and the filtered liquid was concentrated into a thick paste. The remaining *Paeonia lactiflora Pall*. and *Angelica sinensis (Oliv.) Diels* were ground into a fine powder. Then, 20 g of grilled *Glycyrrhiza uralensis Fisch*. was crushed into a fine powder, while the remaining grilled liquorice, *Glycyrrhiza uralensis Fisch*. was boiled in water three times (2 h each time). The decoction was pooled, strained, or left to sit overnight, and the filtrate or supernatant was concentrated to an appropriate amount. This concentrated liquid was mixed with the above thick paste, fine powder, volatile oil, and malt sugar, mixed well, dried, sealed, and stored.

A comprehensive analysis of XYS using UPLC‐Q/TOF‐MS has been described in a prior publication by our research group [35].

#### 2.2.3 Animal Experiments

Male C57BL/6J SPF mice [SYXK (Yue) 2022–0174], weighing 20 ± 2 g, were purchased from Yaokang Biotechnology Co. Ltd. (Guangdong, Foshan). The mice underwent a 7 day adaptation period to adjust to the conditions (5 mice/cage). The experimental conditions were strictly controlled: the room temperature was maintained at 21°C ± 2°C, the relative humidity ranged from 30% to 40%, and a 12 h light/dark cycle was used. We used the GraphPad QuickCalcs randomized tool (https://www.graphpad.com/quickcalcs/randomize1/) for online completely randomized allocation. The mice were randomly allocated to 4 groups: the normal control group (NC), model group (BD), model + XYS group (BD + XYS), and model + Lithium carbonate group (BD + LiCO3). The mouse model was established on the basis of previous reports [18]. The experimental procedure is shown in Figure 5A. This study was approved by the Laboratory Animal Welfare and Ethics Committee of Jinan University (Approval No. IACUC‐20230416‐12), and all procedures adhered to current animal welfare guidelines.

#### 2.2.4 Mouse Models of Bipolar Disorder

##### 2.2.4.1 Stereotaxic Surgery and Virus Injection

First, manic‐like behavior of mice was induced through the injection of adeno‐associated virus (AAV) specific knockdown of Ank3 in CaMKIIα neurons. AAV vectors serotyped with AAV9 coat proteins were packaged by Genechem Co. Ltd. (Shanghai, China). All viral stocks were diluted to a concentration of 1.43 × 10^12 vector genomes per milliliter for injection. The AAV9‐CaMKIIa‐CON540 vector was injected into the NC group, while the model group received AAV9‐CaMKIIa‐Ank3‐RNAi to knock down Ank3 in the hippocampus. The mice were anesthetized with tribromoethanol (0.2 mL/10 g via intraperitoneal injection) and positioned on a stereotactic frame (RWD Life Science, Shenzhen, China). Viral vectors were injected bilaterally into the CA1 region of the hippocampus, at the following coordinates: AP‐2 mm from bregma, ML±1.7 mm, and DV‐1.5 mm from the brain surface. Injections were performed at a controlled rate of 100 nL/min, with 500 nL injected per side. After injection, the capillary was left in place for 5 min to allow the virus to diffuse before being slowly removed. A heating pad was used to facilitate the recovery of the mice from anesthesia. All viral vectors were aliquoted and stored at −80°C. The RNA sequences used for Ank3 knockdown were as follows [34]: shRNA‐Ank3: 5′‐CCGCCTGGTAAAGAGACATAA −3′; shRNA‐CON540: 5′‐TTCTCCGAACGTGTCACGT −3′. At 21 days post‐injection, all the mice underwent behavioral testing to assess their manic‐like behavior.

##### 2.2.4.2 Social Defeat Stress (SDS)

Second, social defeat stress (SDS) was used to induce depression‐like behaviors in mice for 14 days. Male CD‐1 mice (24 weeks old, weighing 40–45 g each) [SYXK (Yue) 2022–0174] were obtained from Weitong Lihua Experimental Animal Technology Co. Ltd. (Guangdong, Foshan). The SDS protocol was conducted as per established guidelines [36]. On the first day of stress, an intruder mouse (from the BD, BD + XYS, and BD + LiCO_3_ groups) was placed in the home cage of a larger, aggressive resident CD‐1 mouse for a 10 min interaction. Following this interaction, a ventilated plastic barrier was installed between the two mice for 24 h. This procedure was repeated daily for 14 days, with the intruder mice rotated to the home cage of a different CD‐1 resident each day before stress testing. After 14 consecutive days of SDS exposure, all the mice underwent behavioral testing to assess their depression‐like behavior.

#### 2.2.5 Behavioral Tests

All behavioral tests were performed under blinded conditions. Specifically, during the experimental testing phase, the animal group was coded and sealed by an independent researcher who did not participate in the behavioral testing. The experimenter who placed the animals into the apparatus and conducted the tests was unaware of the group assignment until all the data collection was completed. In the analysis phase, we used EthoVision XT software to automatically record and quantify all the behavioral parameters.

##### 2.2.5.1 Open Field Test (OFT)

OFT was performed to assess general locomotion and exploratory behavior. We placed each mouse alone in an open field box (50 cm × 50 cm × 40 cm) with a white bottom and set aside 30 min for free exploration and adaptation. The activity of each mouse was recorded by a camera placed above the box. The distance traveled by the mice in the open field box was tracked using EthoVision XT software.

##### 2.2.5.2 Elevated Plus‐Maze Test (EPMT)

The open‐field and enclosed arms of the maze were 30 cm in length and 5 cm in width, respectively. The mice were placed in the center of the maze, looking towards the closed arms, and given the opportunity to freely explore the four arms for 10 min. The experiment was performed under brightly lit ambient conditions. Behavior was recorded with EthoVision XT software, and the time spent in the open arms was analyzed using behavior analysis software.

##### 2.2.5.3 Forced Swim Test (FST)

The FST represents a widely recognized method for evaluating despair behavior in mice. In this test, each mouse was placed individually in a transparent plastic cylinder (25 cm high, 10 cm diameter) filled with water (12 cm deep, 23°C ± 2°C) and permitted to swim for 6 min. The time spent floating without struggling or exercising was measured using EthoVision XT software. A mouse was considered immobile when it floated in the water with minimal movements to keep it from drowning. Experimenters who were unaware of the study conditions recorded the limb immobility time during the last 4 min of the test.

##### 2.2.5.4 Tail Suspension Test (TST)

Murine activity and despair‐related behavior were measured by the TST. The mice were suspended on tape from the apparent belly to the suspension box, 30 cm from the bottom of the suspension, for 6 min. The duration of immobility, defined as the total time spent with only slight movements without making any effort to escape or exercise, was analyzed using EthoVision XT software. The activity of each mouse was specifically recorded during the last 4 min of the test.

##### 2.2.5.5 Social Interaction Test (SIT)

To evaluate the SDS, as in our previous works [37], the SIT was employed. The test was conducted in an open box (42 × 42 cm) featuring an interaction zone, which contained a mesh‐plastic target box (10 × 4.5 cm) and two opposing corner zones. The experiment was performed in two phases: First, the test mouse was placed inside the open arena for 2.5 min without the presence of a social target (no CD1 present). In the second phase, the same mouse was again returned to the open arena for 2.5 min but in the presence of a social target (a novel CD1 mouse). The time spent in the interaction zone during both phases was recorded using a stopwatch. The primary outcome measure was the time ratio, which was calculated by comparing the time spent with the social target relative to the time spent without the social target.

The above behavioral tests were performed in the following fixed order: OFT → SIT → EPMT → TST → FST. Each test was separated by 24 h of rest (animals returned to home cages with ad libitum food and water). All tests were conducted during the light phase (between 9:00 AM and 15:00 PM) under dim light illumination. Animals showing total distance traveled < 500 cm in the open field test or falling off the elevated plus maze were excluded from the corresponding analyses, which was consistent with standard protocols. No animals were excluded from the present study.

#### 2.2.6 Sample Collection

Following the completion of the behavioral tests, the hippocampal tissue samples were carefully harvested. The mice were humanely euthanized, and their brains were promptly excised. Using ophthalmic forceps and scissors, the hippocampus was isolated and immediately immersed in liquid nitrogen to preserve the tissue integrity. The samples were then stored at −80°C for further experiments.

#### 2.2.7 Real‐Time Quantitative Polymerase Chain Reaction (RT‐qPCR)

Total RNA was extracted from the hippocampal tissue and reverse‐transcribed into cDNA using a RevertAid First Strand cDNA Synthesis Kit (Accurate Biology, China, #AG11706). Specific primers for the target genes RAC‐alpha serine/threonine‐protein kinase 1 (AKT1), Interleukin‐6 (IL‐6), Mitogen‐Activated Protein Kinase 3 (MAPK3), Signal Transducer and Activator of Transcription 3 (STAT3), and GAPDH were designed based on the NCBI database (as shown in Table 1) and synthesized by Sangon Biotech Co. Ltd. (Shanghai, China). RT‐qPCR was performed with Power SYBR Green PCR Master Mix (Accurate Biology, China, # AG11701) on a CFX96 Real‐Time System (Bio‐Rad, USA) under the following conditions: 95°C for 30 s, followed by 40 cycles of 95°C for 5 s, and 60°C for 30 s. Relative mRNA expression was quantified using the 2^−ΔΔCT^ method with Bio‐Rad CFX Manager 2.1.

**TABLE 1:** Sequences of Primer sequences used for RT‐qPCR.

Name	Forward primer (5′‐3′)	Reverse primer (5′‐3′)
AKT1	TCAGGATGTGGATCAGCGAGAGTC	AGGCAGCGGATGATAAAGGTGTTG
IL‐6	CTTCTTGGGACTGATGCTGGTGAC	TCTGTTGGGAGTGGTATCCTCTGTG
MAPK3	TTCAGGACCTCATGGAGACAGAC	GAGGAAGTAGCAGATGTGGTCATTG
STAT3	AATCTCAACTTCAGACCCGCCAAC	GCTCCACGATCCTCTCCTCCAG
GAPDH	GGCAAATTCAACGGCACAGTCAAG	TCGCTCCTGGAAGATGGTGATGG

#### 2.2.8 Western Blotting (WB)

Hippocampal tissue was homogenized in radioimmunoprecipitation assay (RIPA) lysis buffer (Beyotime, Shanghai, China) and centrifuged (12,000 rpm for 30 min at 4°C), after which the supernatants were collected. Protein concentrations were determined using a BCA Protein Assay Kit (Beyotime, Beijing, China). Samples (30 μg protein) were subjected to 10% SDS‐PAGE and subsequently transferred to PVDF membranes (Bio‐Rad, Hercules, CA, United States). The membranes were incubated overnight at 4°C with primary antibodies against the following proteins: IL‐6 (1:1000, Abcam), p‐AKT (473) (1:1000, Abmart), AKT (1:1000, Abmart), p‐STAT3 (1:1000, Abmart), STAT3 (1:1000, Proteintech), p‐ERK (1:1000, Abmart), ERK (1:1000, Abmart), and β‐Actin (1:1000, Proteintech). After primary antibody incubation, the membranes were treated with HRP‐conjugated goat anti‐β‐actin secondary antibodies (1:1000; Abcam) for 60 min. The protein bands were quantified using ImageJ software.

#### 2.2.9 Statistical Analysis

Statistical analyses were performed with GraphPad Prism 9.0 (GraphPad Software Inc., USA). The data are presented as the mean ± SEM. All the data were tested for normality using the Shapiro–Wilk test. One‐way analysis of variance (ANOVA) or two‐way ANOVA was used to analyze data from more than two groups, followed by Tukey's multiple comparisons test. A *p* < 0.05 was considered to indicate statistical significance.

## 3 Results

### 3.1 Compounds and Targets of XYS

The biologically active constituents of XYS were retrieved from the TCMSP database. After screening, a total of 161 biologically active constituents from 8 Chinese herbs were identified, including 17 from *Bupleurum Chinense DC*., 2 from *Angelica sinensis (Oliv.) Diels*, 15 from *Smilax glabra Roxb*., 13 from *Paeonia lactiflora Pall*., 7 from *Atractylodes macrocephala Koidz*., 92 from *Glycyrrhiza uralensis Fisch*., 10 from *Mentha canadensis L*., and 5 from *Zingiber officinale Roscoe*. Information on some of the biologically active constituents, specifically the top 30 based on their OB, is presented in Table 2.

**TABLE 2:** The top 30 bioactive components of XYS ranked according to the OB (%).

Drug	MOL ID	Molecule Name	OB (%)	DL
*Radix paeoniae alba*	MOL001918	Paeoniflorgenone	87.59	0.37
	MOL001925	Paeoniflorin_qt	68.18	0.4
	MOL001928	Albiflorin_qt	66.64	0.33
	MOL001910	11alpha, 12alpha‐epoxy‐3beta‐23‐dihydroxy‐30‐norolean‐20‐en‐28,12beta‐olide	64.77	0.38
*Rhizoma atractylodis*	MOL000022	14‐acetyl‐12‐senecioyl‐2E,8Z,10E‐atractylentriol	63.37	0.3
	MOL000020	12‐senecioyl‐2E,8E,10E‐atractylentriol	62.4	0.22
*Herba menthae*	MOL000471	Aloe‐emodin	83.38	0.24
	MOL005190	Eriodictyol	71.79	0.24
*Radix bupleuri*	MOL004644	Sainfuran	79.91	0.23
*Radix glycyrrhizae*	MOL002311	Glycyrol	90.78	0.67
	MOL004990	7,2′,4′‐trihydroxy‐5‐methoxy‐3‐arylcoumarin	83.71	0.27
	MOL004904	Licopyranocoumarin	80.36	0.65
	MOL004891	Shinpterocarpin	80.3	0.73
	MOL005017	Phaseol	78.77	0.58
	MOL004841	Licochalcone B	76.76	0.19
	MOL004810	Glyasperin F	75.84	0.54
	MOL001484	Inermine	75.18	0.54
	MOL000500	Vestitol	74.66	0.21
	MOL005007	Glyasperins M	72.67	0.59
	MOL004941	(2R)‐7‐hydroxy‐2‐(4‐hydroxyphenyl)chroman‐4‐one	71.12	0.18
	MOL004959	1‐Methoxyphaseollidin	69.98	0.64
	MOL000392	Formononetin	69.67	0.21
	MOL004863	3‐(3,4‐dihydroxyphenyl)‐5,7‐dihydroxy‐8‐(3‐methylbut‐2‐enyl)chromone	66.37	0.41
	MOL004903	Liquiritin	65.69	0.74
	MOL004808	Glyasperin B	65.22	0.44
	MOL004829	Glepidotin B	64.46	0.34
	MOL004855	Licoricone	63.58	0.47
	MOL004914	1,3‐dihydroxy‐8,9‐dimethoxy‐6‐benzofurano[3,2‐c]chromenone	62.9	0.53
	MOL004835	Glypallichalcone	61.6	0.19
	MOL004907	Glyzaglabrin	61.07	0.35

According to the TCMSP database, there are 186 components from *Bupleurum Chinense DC*., 51 from *Angelica sinensis (Oliv.) Diels*, 22 from *Smilax glabra Roxb*., 88 from *Paeonia lactiflora Pall*., 18 from *Atractylodes macrocephala Koidz*., 224 from *Glycyrrhiza uralensis Fisch*., 108 from *Mentha canadensis L*., and 52 from *Zingiber officinale Roscoe*. After removing duplicates, 249 unique potential action targets for the biologically active constituents of XYS were identified.

### 3.2 Results of the Search and Screening of BD Disease Targets

A total of 21,361 targets related to BD were identified from the GeneCards database. These targets were analyzed and classified on the basis of their scores, with those exceeding the median score considered potential BD targets. The maximum score among the BD targets was 62.3116, while the minimum score was 0.1394. By using median values of 1.3198, 2.8883, and 5.250, we established 1337 targets with scores greater than 7.946 as potential BD targets. To further enrich our findings, relevant targets from the OMIM, DrugBank, DisGeNET, and TTD databases were integrated, ensuring the removal of duplicates. This process led to the identification of 3294 BD‐associated targets. As illustrated in Figure 1A, a comparison of the targets of the biologically active constituents of XYS with BD disease targets revealed 120 potential XYS targets for BD intervention, which were identified using an online Venn diagram.

**FIGURE 1:**
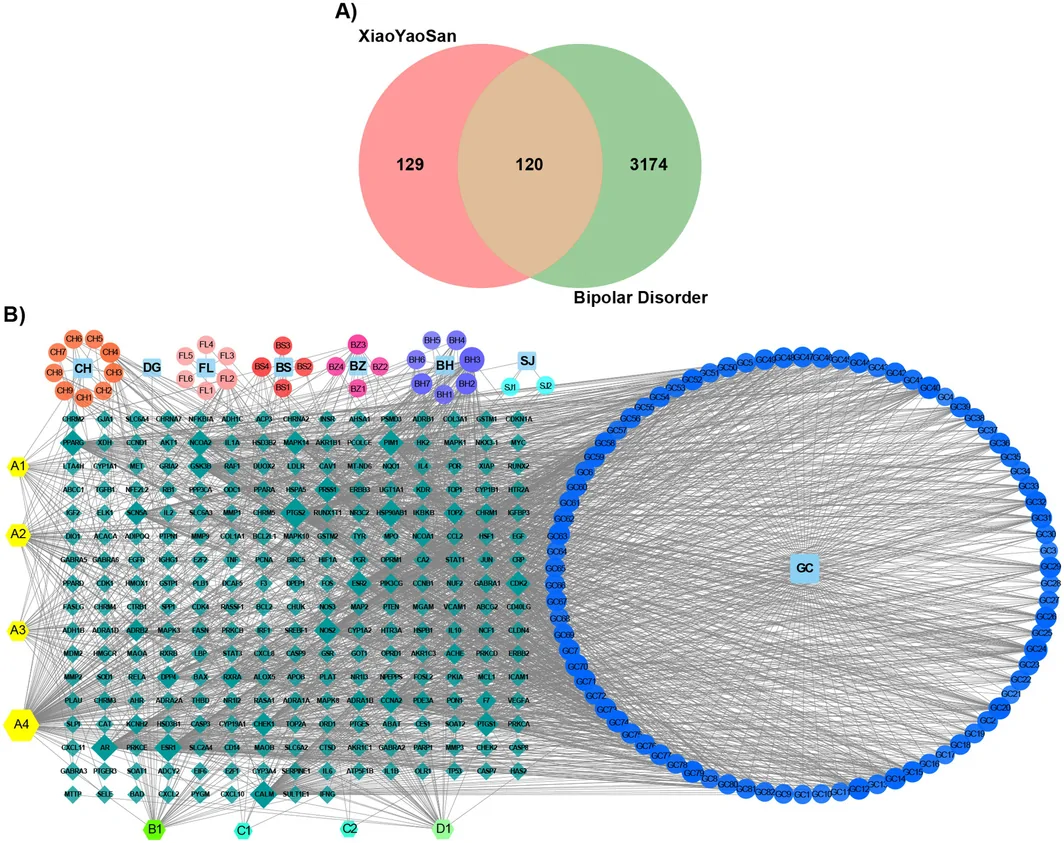
Biologically active constituents and targets of XYS. (A) Venn diagram showing the targets of XYS and BD. (B) “Drugs‐active compounds‐potential targets” network. The hexagons represent the common biologically active constituents of the drugs, the circles represent the biologically active constituents of each drug, and the diamonds represent the predicted targets. The edges represent the interactions between compounds and targets, and the node size is proportional to the degree of interaction. CH stands for *Bupleurum Chinense DC*., DG stands for *Angelica sinensis (Oliv.) Diels*, FL stands for *Smilax glabra Roxb*., BS stands for *Paeonia lactiflora Pall*., BZ stands for *Atractylodes macrocephala Koidz*., BH stands for *Mentha canadensis L*., SJ stands for *Zingiber officinale Roscoe*, and GC stands for *Glycyrrhiza uralensis Fisch*. A1 was common to *Bupleurum Chinense DC*., *Angelica sinensis (Oliv.) Diels*, and *Zingiber officinale Roscoe*; A2 was common to *Bupleurum Chinense DC*., *Paeonia lactiflora Pall*., and *Glycyrrhiza uralensis Fisch*.; A3 and A4 were common to *Bupleurum Chinense DC*. and *Glycyrrhiza uralensis Fisch*.; B1 was common to *Angelica sinensis (Oliv.) Diels*, *Paeonia lactiflora Pall*., and *Zingiber officinale Roscoe*; C1 was common to *Paeonia lactiflora Pall*., *Glycyrrhiza uralensis Fisch*., and *Mentha canadensis L*.; C2 was common to *Paeonia lactiflora Pall*. and *Glycyrrhiza uralensis Fisch*.; and D1 was common to *Glycyrrhiza uralensis Fisch*. and *Mentha canadensis L*.

### 3.3 Drug‐Component‐Target Network of XYS

The relationship network between the biologically active constituents of XYS and their targets was visualized and analyzed using Cytoscape 3.7.2. This analysis revealed a total of 371 nodes, including 249 targets, 122 biologically active constituents, and 2102 relationships, as illustrated in Figure 1B. The degree value was chosen as the primary criterion for analysis, with larger node sizes and darker colors indicating the importance of each node. Cytoscape network analysis indicated that A4 (quercetin) was the core bioactive component of XYS, with a connectivity value of 147, a betweenness of 0.4235, and a closeness of 0.5171. The second most significant bioactive component was A2 (kaempferol), which had a connectivity of 62, a betweenness of 0.0981, and a closeness of 0.4233. GC24 (Licochalcone A) displayed a connectivity of 42, a betweenness of 0.0209, and a closeness of 0.3975. Finally, B1 (beta‐sitosterol) had a connectivity of 39, a betweenness of 0.0473, and a closeness of 0.4008.

### 3.4 PPI Network Construction and Core Target Screening of XYS Intervention in BD

The intersection targets were input into the STRING database under the “Multiple Proteins” section, and “ *Homo sapiens* ” was selected as the species to construct the PPI network for XYS and BD. This PPI network comprised 95 nodes and 307 edges, representing the interaction information among the targets. Additionally, these data were imported into Cytoscape 3.7.2 for visualization of the PPI network. The Degree values of the proteins were calculated using the Network Analyzer function. The results indicated that the 4 targets with the highest DC values were STAT3, AKT1, IL‐6, and MAPK3, with DC values of 0.287, 0.266, 0.234, and 0.223, respectively. Consequently, these four targets were recognized as the core targets of XYS in BD intervention (Figure 2A). Additionally, protein clustering analysis using MCODE identified seven distinct protein clusters (Figure 2B).

**FIGURE 2:**
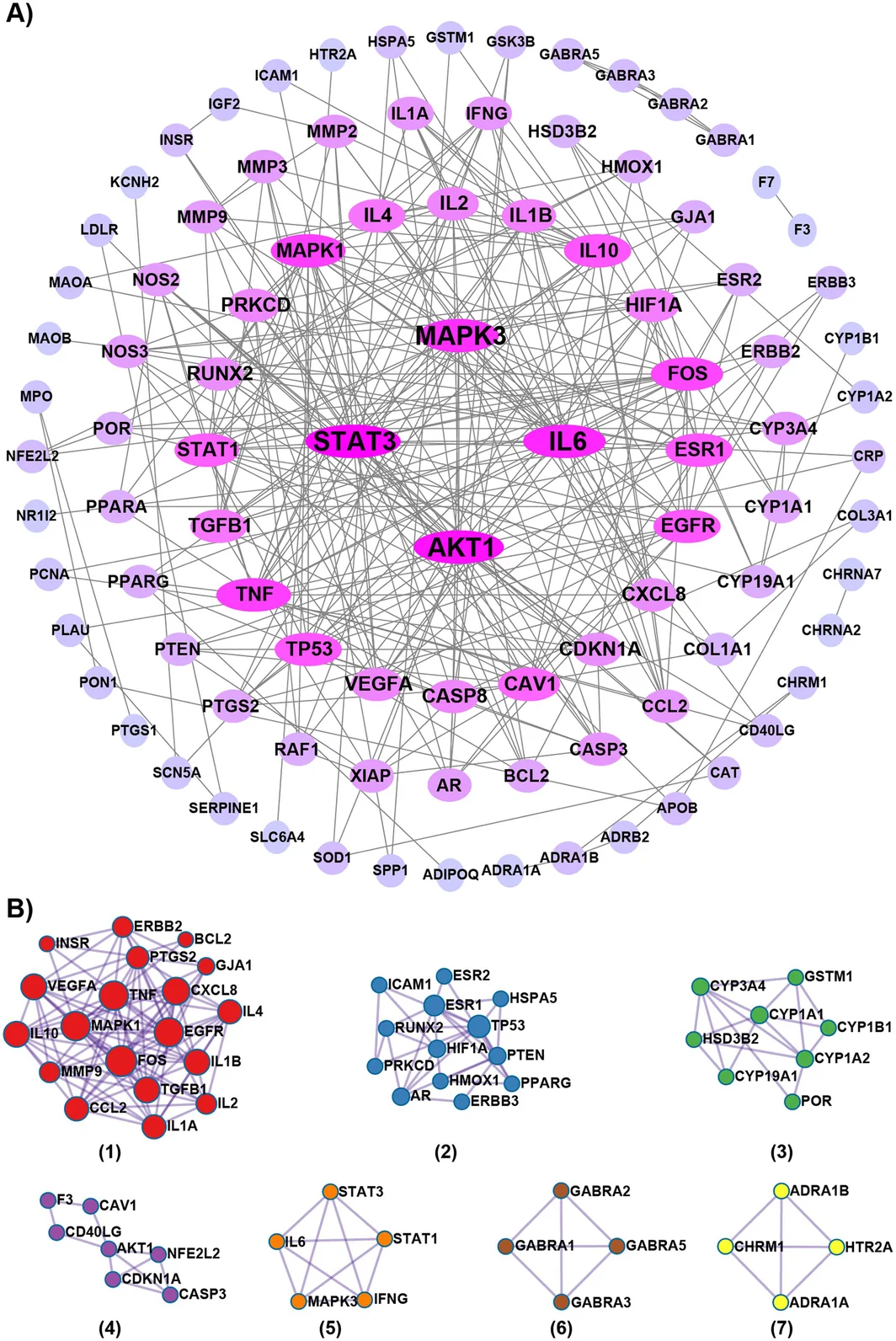
Potential targets identified by PPI analysis. (A) The PPI network. The node size is proportional to the degree value in the network. (B) The seven protein clusters identified by MCODE analysis.

### 3.5 GO Enrichment Analysis

Using the Metascape database platform, GO enrichment analysis was performed on the 95 key nodes. This analysis yielded 1825 GO entries with *p* values of less than 0.05, categorized into 1586 BP, 97 CC, and 142 MF. The results were sorted by log10(*p*) values and visualized using the Weishenxin online platform. The BP, CC, and MF results are presented in a bar chart (Figure 3). In this chart, red bars represent BP, blue bars represent CC, and green bars represent MF, with the horizontal axis on the right indicating the number of key nodes. For BP, the main enriched terms were responses to growth factors, regulation of protein serine/threonine kinase activity, cytokine‐mediated signaling pathway, synaptic transmission, and gamma‐aminobutyric acid signaling pathway. In the CC category, key enriched terms were found in the postsynaptic membrane, mitochondrial intermembrane space, dendrite membrane, GABA receptor complex, and component of the presynaptic membrane. For MF, the primary enriched terms included neurotransmitter receptor activity, protein kinase activity, tumor necrosis factor receptor superfamily binding, GABA‐gated chloride ion channel activity, and inhibitory extracellular ligand‐gated ion channel activity, which are involved in postsynaptic membrane potential regulation.

**FIGURE 3:**
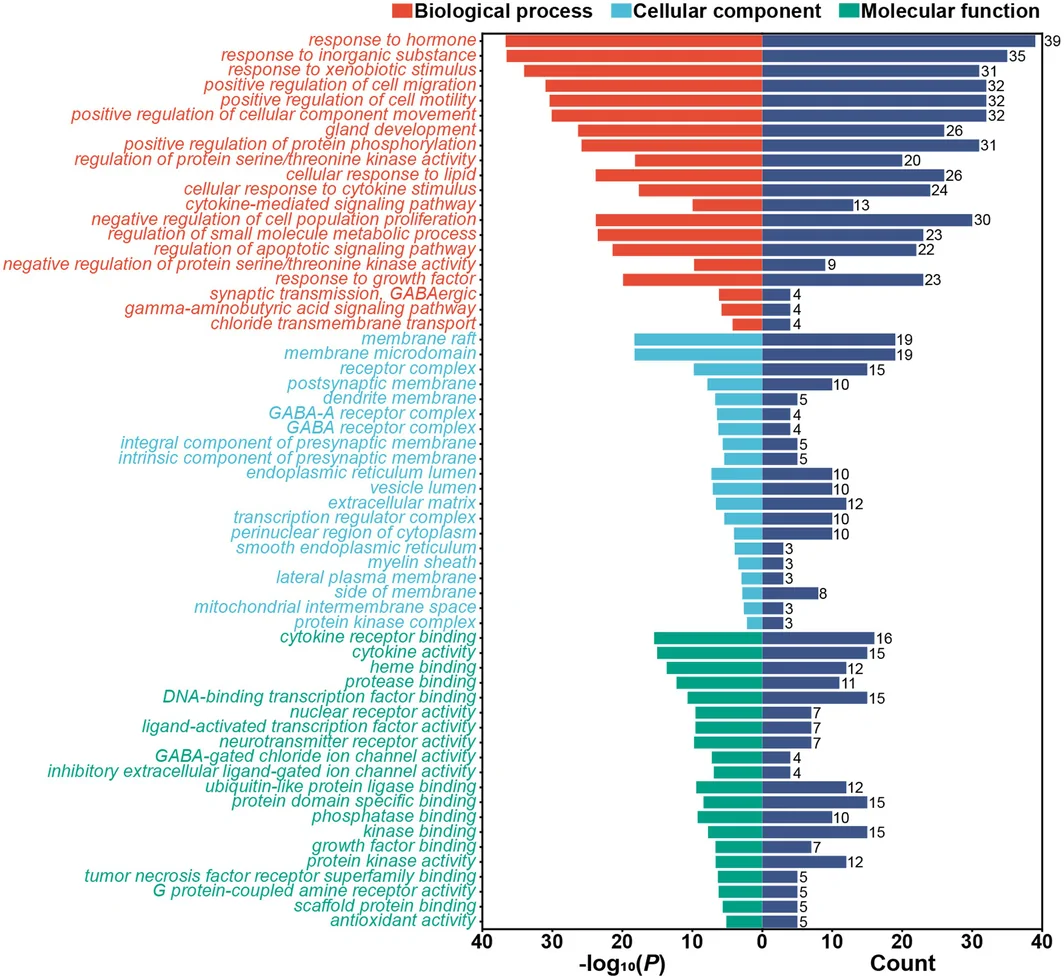
The results of the GO enrichment analysis. In the first 20 enrichment analyses, orange, blue and green represent the enrichment analyses of BP, CC and MF, respectively.

### 3.6 KEGG Pathway Enrichment Analysis

KEGG enrichment analysis was conducted on 95 key nodes through the Metascape platform, resulting in the identification of 186 enriched KEGG pathways with *p* < 0.05. On the basis of the log10(*p*) values, 20 significant KEGG pathways were selected, and a bubble diagram was generated to visually represent the results, as shown in Figure 4A. In the diagram, the color of the bubbles varies from red to green, indicating the log10(*p*) values from smallest to largest. The area of each bubble represents the number of genes linked to the respective pathway, while the horizontal axis represents the degree of gene enrichment within each pathway. Furthermore, each target was categorized according to its corresponding pathway, and the data were imported into Cytoscape 3.7.2 for visual analysis, resulting in the construction of a “pathway‐target” network diagram, as illustrated in Figure 4B. KEGG pathway enrichment analysis revealed that the pathways involved primarily in the effect of XYS ON BD included the IL‐17, HIF‐1, NF‐κB, cGMP‐PKG, and Calcium signaling pathways, among others. A detailed summary of the enriched target pathways is provided in Table 3.

**FIGURE 4:**
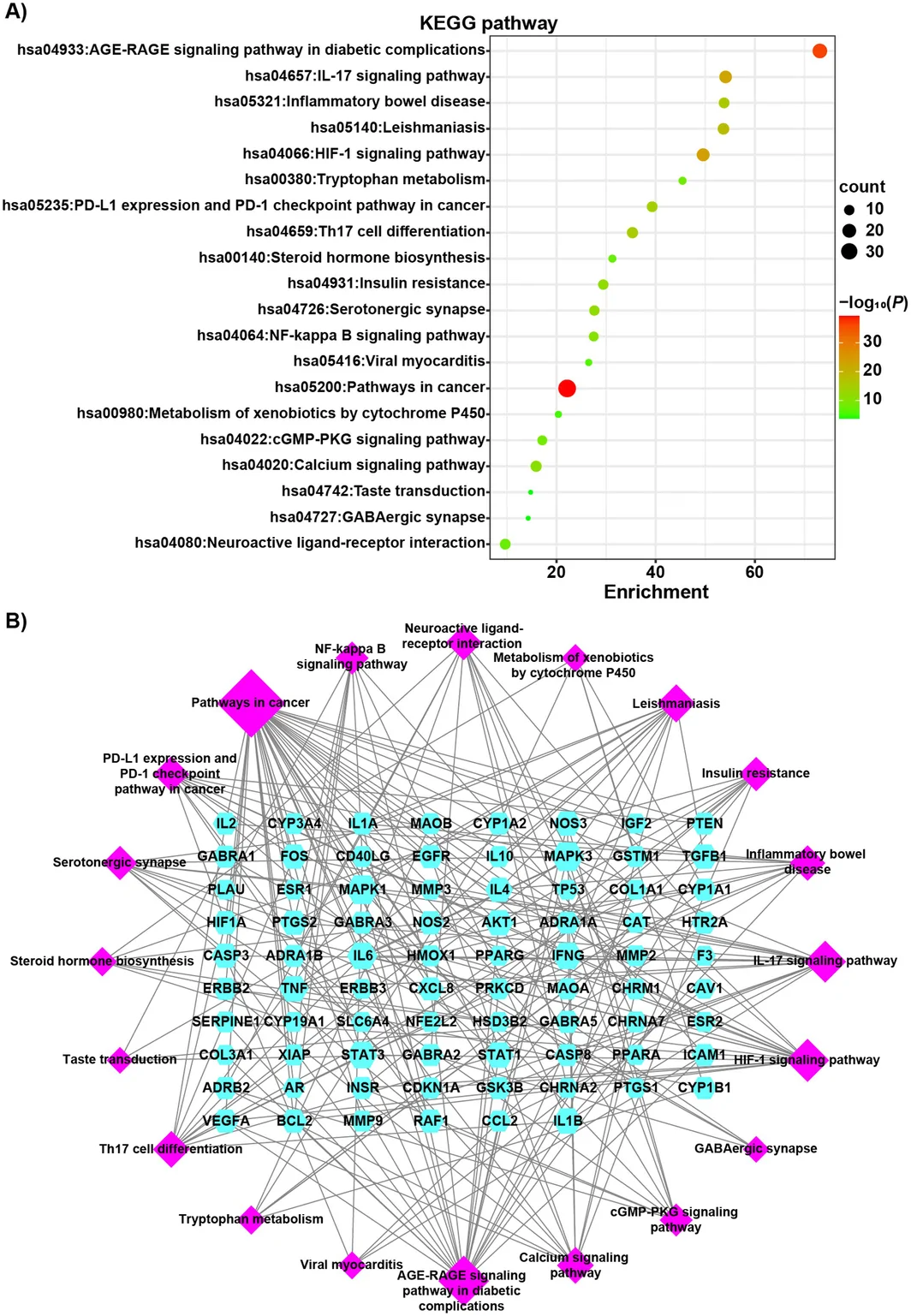
Results of the KEGG enrichment analysis and pathway network. (A) Bubble diagram of the top 20 pathways based on KEGG enrichment analysis. (B) The “target‐pathway” network involved in the mechanism of XYS for BD treatment. The purple diamonds and blue hexagons represent enriched pathways and targets, respectively.

**TABLE 3:** KEGG pathway enrichment analysis of BD targets intervened by XYS.

KEGG	Description	Count	*p*	Hits
hsa05200	Pathways in cancer	37	4.17E‐40	AKT1, XIAP, AR, BCL2, CASP3, CASP8, CDKN1A, EGFR, ERBB2, ESR1, ESR2, FOS, GSK3B, GSTM1, HIF1A, HMOX1, IFNG, IGF2, IL2, IL4, IL6, CXCL8, MMP2, MMP9, NFE2L2, NOS2, PPARG, MAPK1, MAPK3, PTEN,PTGS2, RAF1, STAT1, STAT3, TGFB1, TP53, VEGFA
hsa05235	PD‐L1 expression and PD‐1 checkpoint pathway in cancer	11	4.91E‐15	AKT1, EGFR, FOS, HIF1A, IFNG, MAPK1, MAPK3, PTEN, RAF1, STAT1, STAT3
hsa04933	AGE‐RAGE signaling pathway in diabetic complications	23	3.24E‐37	AKT1, BCL2, CASP3, COL1A1, COL3A1, F3, ICAM1, IL1A, IL1B, IL6, CXCL8, MMP2, NOS3, SERPINE1, PRKCD, MAPK1, MAPK3, CCL2, STAT1, STAT3, TGFB1, TNF, VEGFA
hsa04066	HIF‐1 signaling pathway	17	1.65E‐24	AKT1, BCL2, CDKN1A, EGFR, ERBB2, HIF1A, HMOX1, IFNG, IL6, INSR, NOS2, NOS3, SERPINE1, MAPK1, MAPK3, STAT3, VEGFA
hsa04657	IL‐17 signaling pathway	16	9.18E‐24	CASP3, CASP8, FOS, GSK3B, IFNG, IL1B, IL4, IL6, CXCL8, MMP3, MMP9, MAPK1, MAPK3, PTGS2, CCL2, TNF
hsa05140	Leishmaniasis	13	1.96E‐19	FOS, IFNG, IL1A, IL1B, IL4, IL10, NOS2, MAPK1, MAPK3, PTGS2, STAT1, TGFB1, TNF
hsa04659	Th17 cell differentiation	12	9.97E‐16	FOS, HIF1A, IFNG, IL1B, IL2, IL4, IL6, MAPK1, MAPK3, STAT1, STAT3, TGFB1
hsa05321	Inflammatory bowel disease	11	1.28E‐16	IFNG, IL1A, IL1B, IL2, IL4, IL6, IL10, STAT1, STAT3, TGFB1, TNF
hsa04931	Insulin resistance	10	1.75E‐12	AKT1, GSK3B, IL6, INSR, NOS3, PPARA, PRKCD, PTEN, STAT3, TNF
hsa04726	Serotonergic synapse	10	3.32E‐12	CASP3, HTR2A, MAOA, MAOB, MAPK1, MAPK3, PTGS1, PTGS2, RAF1, SLC6A4
hsa04020	Calcium signaling pathway	12	1.47E‐11	ADRA1B, ADRA1A, ADRB2, CHRM1, CHRNA7, EGFR, ERBB2, ERBB3, HTR2A, NOS2, NOS3, VEGFA
hsa04064	NF‐kappa B signaling pathway	9	4.40E‐11	XIAP, BCL2, CD40LG, ICAM1, IL1B, CXCL8, PLAU, PTGS2, TNF
hsa04022	cGMP‐PKG signaling pathway	9	3.05E‐09	ADRA1B, ADRA1A, ADRB2, AKT1, INSR, NOS3, MAPK1, MAPK3, RAF1
hsa00380	Tryptophan metabolism	6	3.95E‐09	CAT, CYP1A1, CYP1A2, CYP1B1, MAOA, MAOB
hsa00140	Steroid hormone biosynthesis	6	3.99E‐08	CYP1A1, CYP1A2, CYP1B1, CYP3A4, CYP19A1, HSD3B2
hsa00980	Metabolism of xenobiotics by cytochrome P450	5	4.88E‐06	CYP1A1, CYP1A2, CYP1B1, CYP3A4, GSTM1
hsa04080	Neuroactive ligand‐receptor interaction	11	2.01E‐08	ADRA1B, ADRA1A, ADRB2, CHRM1, CHRNA2, CHRNA7, GABRA1, GABRA2, GABRA3, GABRA5, HTR2A
hsa04742	Taste transduction	4	1.60E‐04	GABRA1, GABRA2, GABRA3, GABRA5
hsa04727	GABAergic synapse	4	1.83E‐04	GABRA1, GABRA2, GABRA3, GABRA5
hsa05416	Viral myocarditis	5	1.32E‐06	CASP3, CASP8, CAV1, CD40LG, ICAM1

### 3.7 Effects of XYS on Manic‐Like and Depression‐Like Behavior in BD Mice

To evaluate the effects of XYS on BD‐like behavior induced by Ank3 knockdown combined with CSDS, mice were subjected to AAV‐mediated Ank3 knockdown (except those in the NC group). XYS and LiCO_3_ interventions were administered 7 days after AAV injection. Manic‐like behavior was assessed 3 weeks after AAV injection using the OFT, EPMT, SIT, TST, and FST (Figure 5A). The results indicated that XYS effectively reduced both the total movement distance and the average movement distance per 5 min in the OFT (Figure 5B–D). Additionally, while XYS treatment reduced the residence time in the open arm during the manic phase in BD mice, this decrease was not significant (Figure 5E,F). Furthermore, during the manic phase, XYS treatment significantly increased immobility time in BD mice in both the TST and the FST (Figure 5G,H), suggesting that XYS effectively alleviated manic‐like behavior in BD mice. Notably, the social interaction ratio for all the groups exceeded 1 (Figure 5I), indicating that all the groups exhibited normal social behavior.

**FIGURE 5:**
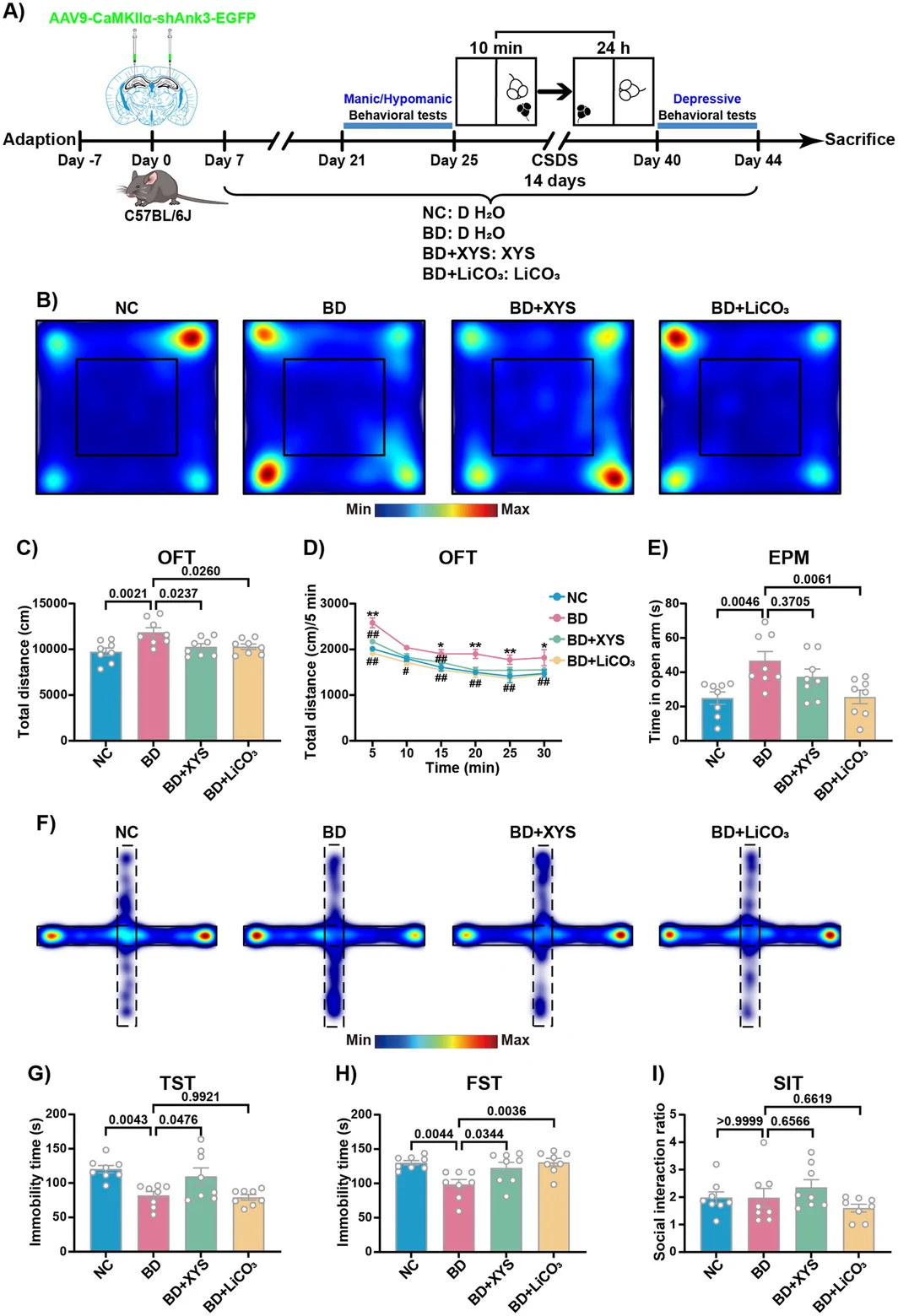
Effect of XYS on the activities of BD mice with manic‐like behaviors. (A) Timeline of the experimental process. (B) Heatmap of OFT, (C) total distance traveled and (D) average distance traveled per 5 min in each group of mice. (E) Residence time in the open arm of EPMT and (F) heatmap. Immobility time of mice in the (G) TST and (H) FST. (I) The ratio of social interaction time. The data are presented as the mean ± SEM (*n* = 8). *p < 0.05, **p < 0.01 vs. the NC group; #*p* < 0.05, ##*p* < 0.01 vs. the BD group. NC: Control group; BD: Model group; BD + XYS: Model + Xiaoyaosan group; BD + LiCO_3_: Model + Lithium carbonate group.

To assess the effect of XYS on depressive‐like behavior in BD mice after 14 days of CSDS, we performed the same behavioral tests used to evaluate manic‐like behavior. The results revealed that XYS significantly increased both the total movement distance and the average movement distance per 5 min from 0 to 5 min and from 20 to 25 min in the OFT (Figure 6A–C). In addition, XYS significantly increased the residence time of BD mice in the open arm (Figure 6D,E) and substantially improved the SIT ratio of BD mice during the depression phase (Figure 6F,G). Moreover, XYS reduced the immobility time of BD mice during the depression phase in the TST and FST (Figure 6H,I). These findings demonstrated that XYS significantly improved both manic‐like and depression‐like behavioral symptoms in BD mice.

**FIGURE 6:**
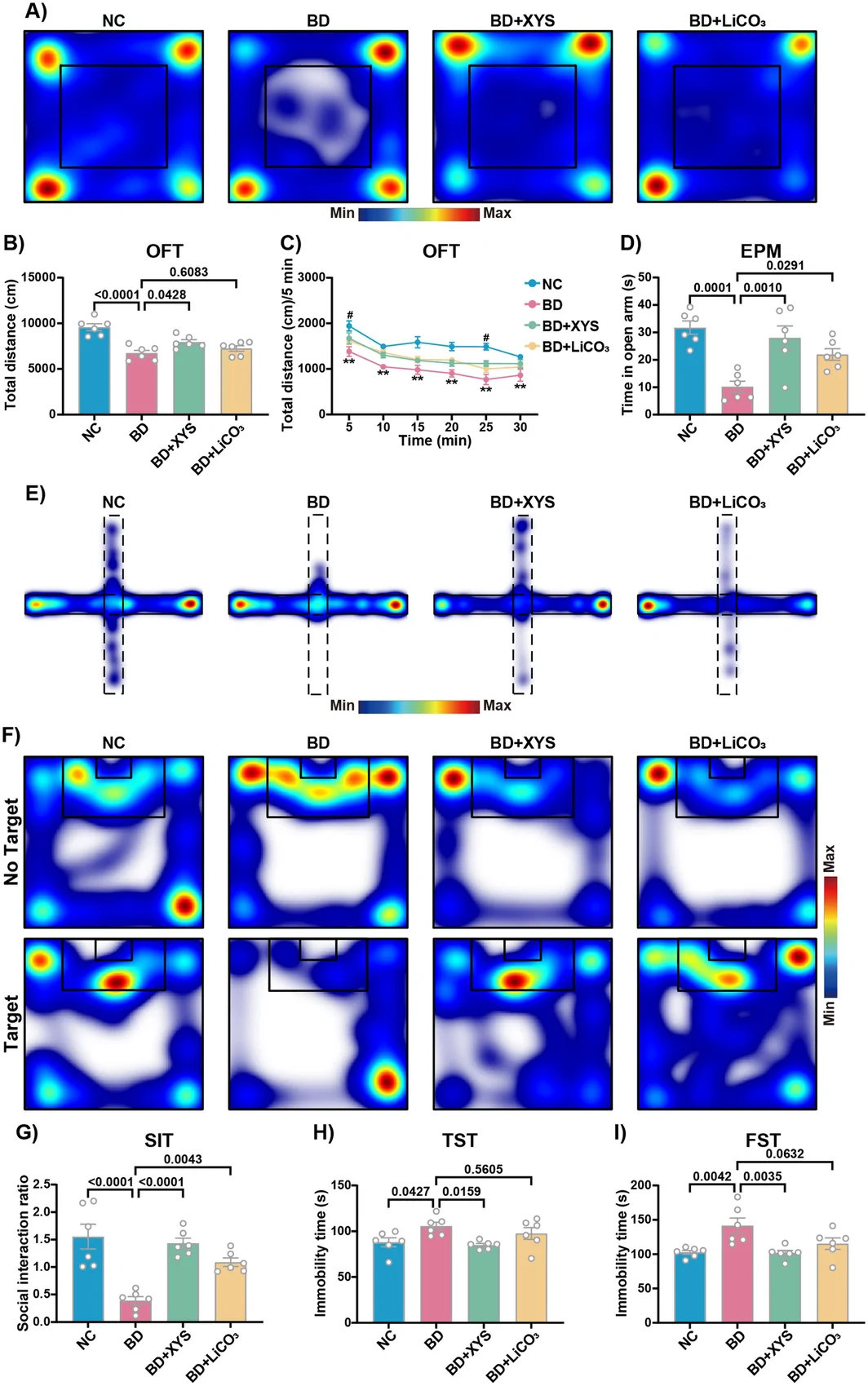
Effect of XYS on the activities of BD mice with depression‐like behaviors. (A) Heatmap of OFT, (B) total distance traveled and (C) average distance traveled per 5 min in each group of mice. (D) Residence time in the open arm of EPMT and (E) heatmap. (F) Heatmap of social interaction and (G) the ratio of social interaction time. Immobility time of mice in the (H) TST and (I) FST. The data are presented as the mean ± SEM (*n* = 6). **p < 0.01 vs. the NC group; #*p* < 0.05 vs. the BD group. NC: Control group; BD: Model group; BD + XYS: Model + Xiaoyaosan group; BD + LiCO_3_: Model + Lithium carbonate group.

### 3.8 Effects of XYS on Potential Targets in BD Mice

To investigate the anti‐BD mechanism of XYS, we assessed its effects on the expression of potential targets of STAT3, AKT1, IL‐6, and MAPK3 signaling using RT‐qPCR and WB. XYS significantly reduced the relative mRNA levels of STAT3, AKT1, IL‐6, and MAPK3 in the hippocampus of BD mice (Figure 7A–D). Additionally, increases in relative hippocampal protein levels of p‐STAT3, p‐AKT(Ser473), and IL‐6 in the BD group were attenuated by XYS treatment, which also reduced p‐ERK expression (Figure 7E–L). These findings suggested that STAT3, AKT, and IL‐6 might serve as core targets of XYS in the treatment of BD.

**FIGURE 7:**
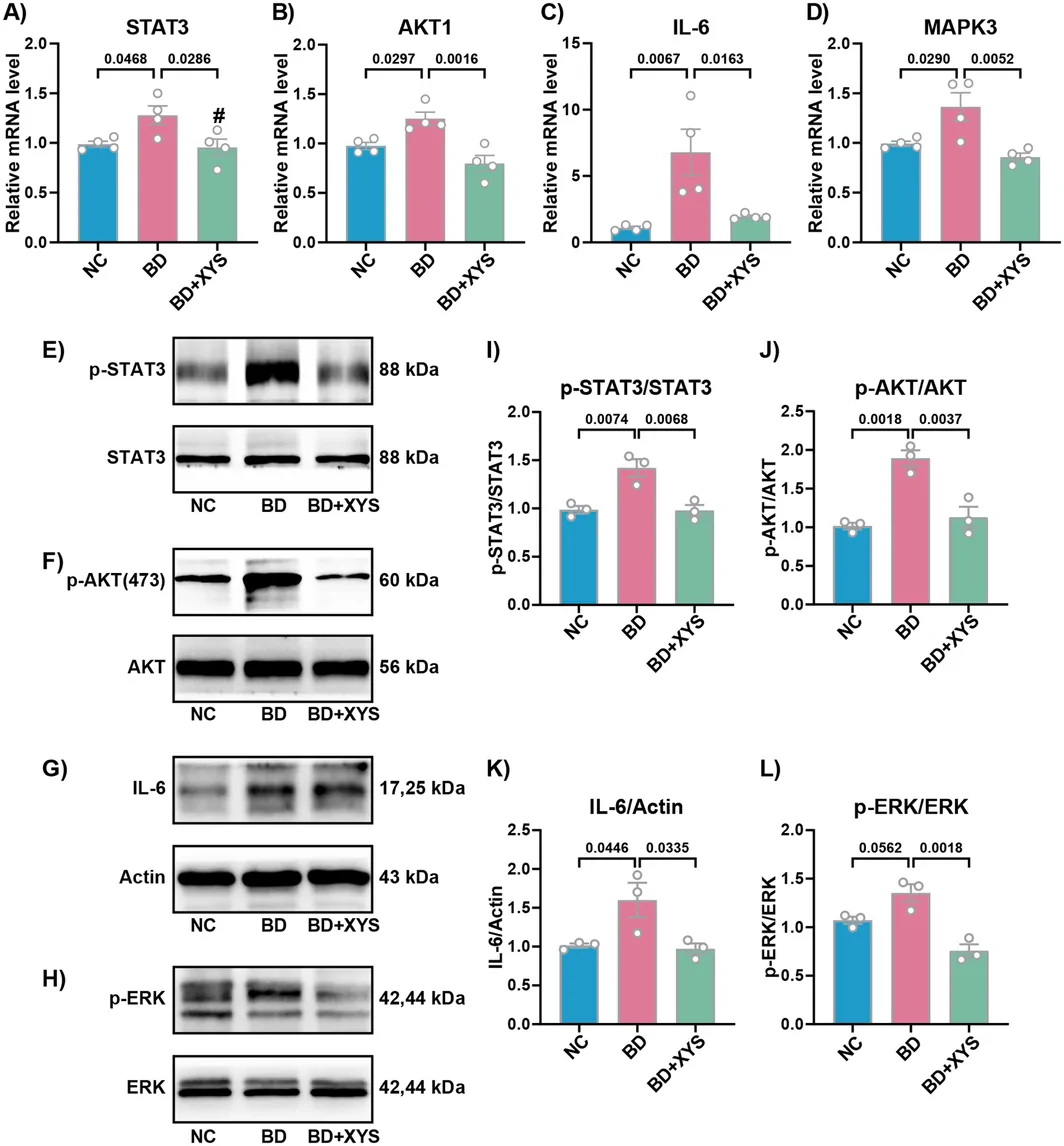
Effects of XYS on potential targets in BD mice. (A–D) The mRNA levels of STAT3, AKT1, IL‐6, and MAPK3 in the hippocampus of BD mice were determined by RT–qPCR. The data are presented as the mean ± SEM (*n* = 4). (E–L) The protein levels of p‐STAT3, p‐AKT(473), IL‐6, and p‐ERK were determined by Western blotting and quantified by densitometry. The data are presented as the mean ± SEM (*n* = 3). NC: Control group; BD: Model group; BD + XYS: Model + Xiaoyaosan group.

### 3.9 Molecular Docking

Molecular docking was performed to validate the predicted interactions between the biologically active components of XYS, including quercetin, kaempferol, Licochalcone A, and beta‐sitosterol, along with the core targets STAT3, AKT1, IL‐6, and MAPK3. It is generally accepted that the stability of the binding conformation is inversely related to the binding energy, meaning that a lower binding energy indicates a higher probability of the protein binding to the compound. To clearly understand the interactions between bioactive compounds and target proteins, the comprehensive molecular binding energies are shown in Table 4, and partial molecular docking conformation diagrams are shown in Figure 8. The results indicated that kaempferol exhibited the most stable binding with the core target STAT3 (binding energy of −7 kcal·mol−1). Beta‐sitosterol showed the most stable binding with AKT1 (binding energy of −6.9 kcal·mol−1). Quercetin had the most stable binding with the target IL‐6 (binding energy of −7.1 kcal·mol−1). Finally, for Licochalcone A, the most stable binding was observed with MAPK3 (binding energy of −9 kcal·mol−1).

**TABLE 4:** Binding energy table of active constituents and core targets.

Active Constituents	Binding Energy/(kcal·mol‐1)			
	AKT1	IL‐6	STAT3	MAPK3
Beta‐sitosterol	−6.9	−6.7	−7.1	−9.8
Quercetin	−6.1	−7.1	−6.9	−9.1
Kaempferol	−6	−6.5	−7	−9
Licochalcone A	−6.2	−6.7	−6.8	−9

**FIGURE 8:**
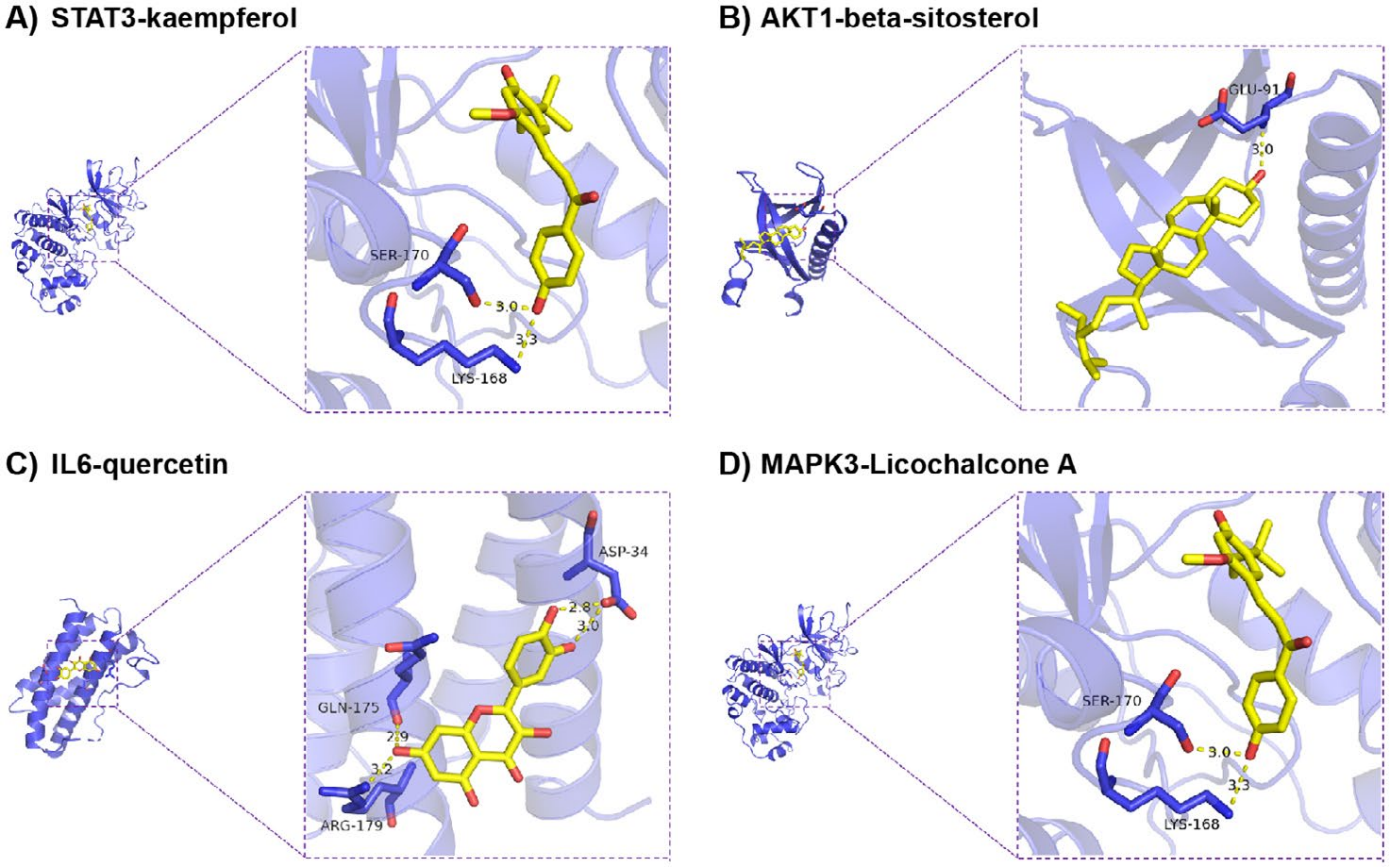
Conformation diagrams of partial molecular docking. (A) Kaempferol and STAT3, (B) beta‐sitosterol and AKT1, (C) quercetin and IL‐6, and (D) Licochalcone A and MAPK3.

## 4 Discussion

Network pharmacology, an emerging discipline centred on network targets, encompasses biological network modules such as biologically active constituents of TCM, targets of action, diseases, and multiple pathways [38]. This method is a powerful tool for studying TCM compounds. TCM compounds have relatively complex biological active ingredients that produce pharmacological effects via multiple drug components, targets, signaling pathways and other mechanisms. For example, Haiyu Xu et al. [39] used network pharmacology to predict the key biologically active constituents and targets of Huashi Baidu Decoction in the treatment of COVID‐19. Similarly, Network pharmacology technology was used by Gu Yang et al. [40] to analyze the effective components and key signaling pathways of Sinisan in the treatment of sepsis. XYS is a TCM prescription composed of eight Chinese herbal medicines. Both complete prescriptions and individual herbs exert antidepressant, anti‐inflammatory and other therapeutic effects through multiple signaling pathways [41, 42]. Categories of compound ingredients of this prescription include volatile oils, alkaloids, flavonoids, saponins, polysaccharides, and sterols, among others [35]. Our research group has reported that XYS can achieve antianxiety and antidepressant effects through the regulation of various bioactive components and pathways on the basis of network pharmacology [35]. In the current study, network pharmacology was first used to predict of the potential targets of XYS in BD treatment. The core bioactive components of XYS include kaempferol, β‐sitosterol, quercetin, and Licoricechalcone A. Kaempferol, quercetin, and Licochalcone A belong to the flavonoid compounds and are derived from herbs such as *Bupleurum Chinense DC., Paeonia lactiflora Pall., Glycyrrhiza uralensis Fisch., and Zingiber officinale Roscoe* in XYS. Flavonoids are known for their antioxidant, anti‐inflammatory, antiviral, and antibacterial effects, which are mediated through the brain‐gut axis pathway [43]. Both kaempferol and quercetin exhibit significant anti‐inflammatory and antioxidant effects in neurosystem‐related diseases [44, 45]. Moreover, β‐sitosterol is a sterol compound and is derived from the herbs composing XYS: *Paeonia lactiflora Pall., Angelica sinensis (Oliv.) Diels, and Zingiber officinale Roscoe* in XYS. Studies have demonstrated that β‐sitosterol can cross the blood–brain barrier and possesses anti‐inflammatory and antidepressive properties [46]. At the intersection between the potential targets of XYS and BD, we identified 120 potential targets, with the top four core active targets being STAT3, AKT1, IL‐6, and MAPK3. These findings indicated that the bioactive compounds identified through network pharmacology studies contributed significantly to the anti‐inflammatory and antipsychiatric effects of XYS and might represent the core biologically active constituents responsible for its therapeutic efficacy.

BD is a common psychiatric illness with high prevalence and heritability. Despite its high degree of heritability, gene and environmental interactions are commonly accepted as the major contributing factors to BD pathogenesis. The Ank3 gene mutation is associated with BD in patients. In rodents, Shanshan Zhu et al. [18] successfully modeled the behavioral phenotype of BD by knocking out the Ank3 gene in CaMKIIa neurons in the mouse brain in combination with CSDS. The ventral hippocampus is a widely expressed region that governs emotions, stress, and behavior [47, 48]. Furthermore, increased hippocampal neuronal activity has been documented previously in these animal models [49, 50]. Therefore, in our current study, we used stereotaxic surgery and virus injection of AAV‐shAnk3 into the hippocampus to specifically knock down the Ank3 gene in CaMKIIa neurons, and the mice (AAV‐shAnk3) exhibited significant manic‐like behavior after 3 weeks of behavioral testing. For example, the total distance traveled in the OFT and the residence time in the open arm of BD mice during the manic phase significantly increased, whereas the immobility time in the TST and FST significantly decreased. These findings suggested that hippocampus‐specific Ank3 gene knockdown induced manic‐like behavior in mice. CSDS was subsequently applied to these Ank3 gene knockdown mice to trigger a behavioral transition from a manic‐like state to a depressive‐like state. After 2 weeks of CSDS, the mice exhibited significant depressive‐like behaviors. Specifically, the total distance traveled in the OFT and the residence time in the open arm significantly decreased during the depressive phase. Furthermore, compared with those in the NC group, the immobility duration of BD mice in the TST and FST increased, and their social interaction decreased. These results are in accordance with those of earlier studies [18], indicating that Ank3 gene knockdown combined with CSDS can successfully induce a BD model from mania to depression in mice. More importantly, on the basis of this animal model, these behaviors were significantly reversed by XYS, indicating that XYS may have a positive effect on the treatment of BD.

After confirming the significant anti‐BD effect of XYS, elucidating its mechanism has become key to restricting its clinical application. Numerous successful applications of methods combining network pharmacology, molecular docking, and experimental validation in interpreting the mechanism of action of TCM and its active ingredients have been reported [51, 52]. Therefore, in this study, we further comprehensively applied network pharmacology, molecular docking, and experimental verification methods to elucidate the specific mechanism underlying the anti‐BD activity of XYS at the mRNA and protein levels. Furthermore, PPI network analysis of network pharmacology revealed the core targets of XYS against BD, including STAT3, AKT1, IL‐6, and MAPK3. The role of the inflammatory response has emerged in BD [53, 54, 55]. Subsequent experimental validation revealed that both high expression levels of STAT3, AKT1, IL‐6, and MAPK3 in BD mice and low expression content were reversed by XYS, which further validated the results of network pharmacology analysis. As far as each core target is concerned, STAT3 is a member of the STAT family of proteins and plays a crucial role in cellular signal transduction processes, with a signal transducer and activator of transcription. Abnormal activation of STAT3 has been associated with various diseases [56], including inflammatory diseases, autoimmune diseases, and cancers [57, 58]. AKT1 is a serine/threonine kinase that belongs to the AKT family and plays a role in cellular processes, and overactivation of AKT1 can reduce the synthesis of peroxidases, thereby affecting the inflammatory response [59]. IL‐6 is an important inflammatory mediator involved in various physiological processes, such as immune responses, inflammation, hematopoiesis, bone metabolism, and embryonic development [60]. The elevated production of IL‐6 during tissue damage or infection therefore promotes the inflammatory response. MAPK3, which includes Extracellular Signal‐Regulated Kinase 1 (ERK1), is a serine/threonine protein kinase that belongs to the mitogen‐activated protein kinase (MAPK) family. Our research further revealed that the potential targets associated with the intersection of XYS and BD were functionally enriched in inflammation‐related pathways, such as the IL‐17, HIF‐1, and NF‐κB signaling pathways. Thus, the present findings demonstrated that the therapeutic effect of XYS in the treatment of BD was closely associated with the regulation of inflammatory pathways and involved important targets, such as STAT3, AKT1, IL‐6, and MAPK3. Therefore, a molecular docking approach involving computer‐aided verification was used to further validate the prediction via network pharmacology analysis. The binding energy from molecular docking is usually calculated to evaluate the degree of affinity of ingredients with protein targets [61]. Our molecular docking results indicated that the average binding energy between the core bioactive component of XYS and the core targets predicted by network pharmacology ranged from −6.0 to −9.8 kcal/mol. The results suggested that the core bioactive component of XYS could bind well to the active sites of protein targets.

Although we predicted the potential targets at the intersection of XYS and BD through network pharmacology and experimentally validated the core biologically active constituents and their potential targets in the treatment of BD, several limitations remain. We did not conduct in vivo experimental validation of the therapeutic effects of the four core biologically active constituents—kaempferol, β‐sitosterol, quercetin, and Licochalcone A—on BD. Such confirmatory analyses require further verification in future studies.

## 5 Conclusion

XYS is involved in multi‐component, multi‐target, and multi‐pathway treatment, offering potential therapeutic benefits in the management of BD. Modulation of core targets such as STAT3, AKT1, IL‐6, and MAPK3 may exert significant anti‐inflammatory effects. These findings establish a new foundation for exploring additional mechanisms of action for XYS in the prevention and treatment of BD.

## Author Contributions

Man Chen and Nana Ding writing – review and editing, writing – original draft, validation, conceptualization, methodology, data curation. Xingmin Qiu: data curation, writing – review and editing. Lan Li: methodology, validation, data curation. Yali Guo: methodology, data curation. Rongyanqi Wang: methodology, formal analysis. Xiaowei Mo: formal analysis. Tan Zou: methodology. Shangzhen Yu: supervision. Xiaojuan Li: review and editing, conceptualization, funding acquisition, project administration, supervision. Jiaxu Chen: review and editing, conceptualization, funding acquisition, project administration, supervision.

## Ethics Statement

The Animal Experiment Protocol listed below has been reviewed and approved by the Laboratory Animal Welfare and Ethics Committee of Jinan University (Approval No. IACUC‐20230416‐12).

## Conflicts of Interest

The authors declare no conflicts of interest.

## Data Availability

The data that support the findings of this study are available from the corresponding author upon reasonable request.
